# RESCUE: An end-to-end multi-agent LLM system for proactive rare-disease patient screening in the EHR

**DOI:** 10.64898/2026.06.24.26356357

**Published:** 2026-07-01

**Authors:** Cong Liu, Alexa Geltzeiler, Adam Afyouni, Mengshu Nie, Kamalakkannan Ravi, Courtney French, Wendy Chung

## Abstract

**Background:**

Rare diseases affect a significant portion of the global population, yet patients often endure a lengthy diagnostic odyssey, frequently missing the opportunity for timely whole-exome or whole-genome sequencing (WES/WGS). Existing informatics tools often rely on pre-identified patients or rigid, institution-specific rule sets, failing to address the broader operational question of clinical necessity and feasibility.

**Method:**

We introduce RESCUE, an end-to-end, multi-agent LLM-powered workflow designed for proactive rare-disease screening across the entire electronic health record (EHR). RESCUE utilizes a team of specialized agents including Ontology, Modeling, Screening, and Review, to automate the screening process. The Ontology Agent classifies clinical data into a four-tier genetic-evidence taxonomy; the Modeling Agent builds a positive-unlabeled (PU) XGBoost classifier to identify potential cases; the Screening Agent applies these models across the EHR population; and the Review Agent evaluates candidates by sampling clinical notes to ensure medical necessity and operational feasibility for sequencing.

**Results:**

Our retrospective evaluation on a holdout set (n=12,591) demonstrated strong discrimination (AUC 0.808). Among 175,842 eligible patients from an institutional base of ~494,577, RESCUE-flagged candidates were 7.4-fold more likely to receive subsequent genetic workups compared to controls. Blinded manual chart reviews confirmed that RESCUE identifies previously missed, medically necessary patients with 80% precision, while simultaneously accounting for prior testing history.

**Conclusion:**

By decoupling expert roles into modular agents, RESCUE offers a flexible, scalable, and adaptable framework for rare-disease screening. This approach overcomes the limitations of traditional rule-based methods and provides a reproducible, agentic pathway to reduce diagnostic delays and improve patient care at an institutional scale.

## Full Text Availability

The license terms selected by the author(s) for this preprint version do not permit archiving in PMC. The full text is available from the preprint server.

