# RESCUE: An end-to-end multi-agent LLM system for proactive rare-disease patient screening in the EHR

**DOI:** 10.64898/2026.06.24.26356357

**Published:** 2026-08-07

**Authors:** Cong Liu, Alexa Geltzeiler, Adam Afyouni, Mengshu Nie, Kamalakkannan Ravi, Courtney French, Monica Wojcik, Wendy K. Chung

**Affiliations:** 1 Boston Children’s Hospital, Department of Pediatrics, Division of Genetics and Genomics, Boston, MA; 2 Boston Children’s Hospital, Children’s Hospital Informatics Program, Boston, MA; 3 Harvard Medical School, Department of Pediatrics, Boston, MA; 4 Boston Children’s Hospital, Information Services Department, Research Computing Group; 5 Boston Children’s Hospital, Department of Pediatrics, Division of Newborn Medicine & Genetics and Genomics

## Abstract

**Background:**

Rare diseases affect a significant portion of the global population, yet patients often endure a lengthy diagnostic odyssey, frequently missing the opportunity for timely diagnoses with exome or genome sequencing (ES/GS). Existing informatics tools often rely on pre-identified patients or rigid, institution-specific rule sets, failing to address the broader operational question of clinical utility and feasibility.

**Methods:**

We introduce RESCUE (*Rare Disease Detection and Escalation Support via a Learning Health System*), an end-to-end, multi-agent LLM-powered workflow designed for proactive rare-disease diagnosis across the entire electronic health record (EHR). RESCUE utilizes a team of specialized agents including Ontology, Modeling, Screening, and Review, to automate the screening process to identify candidates for diagnostic testing based on their clinical features. The Ontology Agent classifies clinical data into a four-tier genetic-evidence taxonomy; the Modeling Agent builds a positive-unlabeled (PU) XGBoost classifier to identify potential cases; the Screening Agent applies these models across the EHR population; and the Review Agent evaluates candidates by sampling clinical notes to ensure medical necessity and operational feasibility for genomic testing.

**Results:**

Using electronic medical record data from a pediatric hospital, our retrospective evaluation on a holdout set (n=12,591) demonstrates strong discrimination between patients who received diagnostic genomic testing and those who did not (AUC 0.808). Of nearly 500,000 patients in the institutional base, 175,842 met inclusion criteria for screening; among these, RESCUE-flagged candidates were 7.4-fold more likely to receive subsequent genomic assessments compared to controls. Blinded manual chart reviews confirmed that RESCUE identifies previously missed, medically appropriate patients for ES/GS with 80% precision, while simultaneously accounting for prior testing history.

**Conclusions:**

By decoupling expert roles into modular agents, RESCUE offers a flexible, scalable, and adaptable framework for screening patients for rare-disease diagnostic genomic testing. This approach overcomes the limitations of traditional rule-based methods and provides a reproducible, agentic pathway to reduce diagnostic delays and improve patient care at an institutional scale.

## Description

RESCUE is a multi-agent LLM system that screens an entire hospital EHR to identify patients who may benefit from diagnostic exome or genome sequencing. In a retrospective evaluation, RESCUE-flagged candidates were 7.4-fold more likely to receive subsequent genomic assessments, and blinded chart review confirmed that 80% were medically appropriate and previously unrecognized.

## 1. Background

Rare diseases collectively affect 3.5~6% of the population, spanning more than 7,000 distinct disorders, and most are genetic in origin^1^. For suspected Mendelian diseases, exome and genome sequencing (ES/GS) yield molecular diagnosis in 25–50% of suspected cases^2–4^, and ES/GS is recommended as a first or second-tier test for congenital anomalies, developmental delay, intellectual disability, and many other indications^5,6^. Even so, the average time between first symptoms and a diagnosis is 5–7 years^7^, and most who could benefit are never referred to a clinical geneticist^8^. Together, these observations point to a recognition gap rather than a testing gap, where the limiting step is identifying which patients should be offered genomic sequencing.

Most existing tools assume the patient has already been identified. Ontology-based pipelines^9,10^ and gene-prioritization tools such as Phenomizer, Phenolyzer, Exomiser, Phen2Gene, Phen2Disease^11,12^, PheNet^13^, and AMELIE^14–17^ all start from a phenotype list a clinician has already assembled for a patient who is undergoing analysis. Recently, ThinkRare^18^ scans the EHR with a hand-crafted rule set, but its rules are tied to one institution’s coding taxonomy and do not easily transfer to other sites. Furthermore, none of these tools address the essential operational question: would this patient benefit from genomic testing, has the diagnostic work-up already been completed, and has the patient been assess by a geneticist?

Large language models (LLMs) provide the potential to change the landscape. LLM can now read clinical notes at close to expert level^19^, and extract phenotype concepts out of free text^20,21^. Additionally, they can act as *agents:* calling tools like database queries, code execution, and model training^22,23^, review charts to carry out multi-step tasks. This agentic capability is a good fit for EHR based rare-disease screening, where the workflow draws on many pre-validated tools and must interact with several separate digital systems.

In this study we introduce **RESCUE** (*Rare Disease Detection and Escalation Support via a Learning Health System*), an EHR-screening *agentic workflow* for proactive identification of patients likely to have rare diseases who would benefit from clinical genomic sequencing. RESCUE is a multi-agent system consisting of multiple LLM-powered agents, each with a skill inventory including free-text description and pre-validated python script. To our knowledge, RESCUE is the first agentic workflow to run end to end at EHR scale: build the cohort, train the model, screen with both trained model and rules, and review charts with operational constraints.

## 2. Methods

### Study setting

RESCUE was developed and evaluated using the i2b2 enterprise data warehouse^24^ (hosted on Snowflake) of a large tertiary pediatric hospital. Each agent is powered by a customized OpenAI Codex CLI, with all LLM calls directed to a HIPAA-compliant intramural Azure OpenAI endpoint. To ensure patient privacy, tracing is disabled, and the Codex’s web browsing capabilities are turned off to prevent accidentally sending protected health information (PHI) to external sources. By employing these measures, no PHI leaves the institutional firewall. This study has been approved by the Boston Children’s Hospital institutional review board.

#### Agent architecture

**Figure 1.**
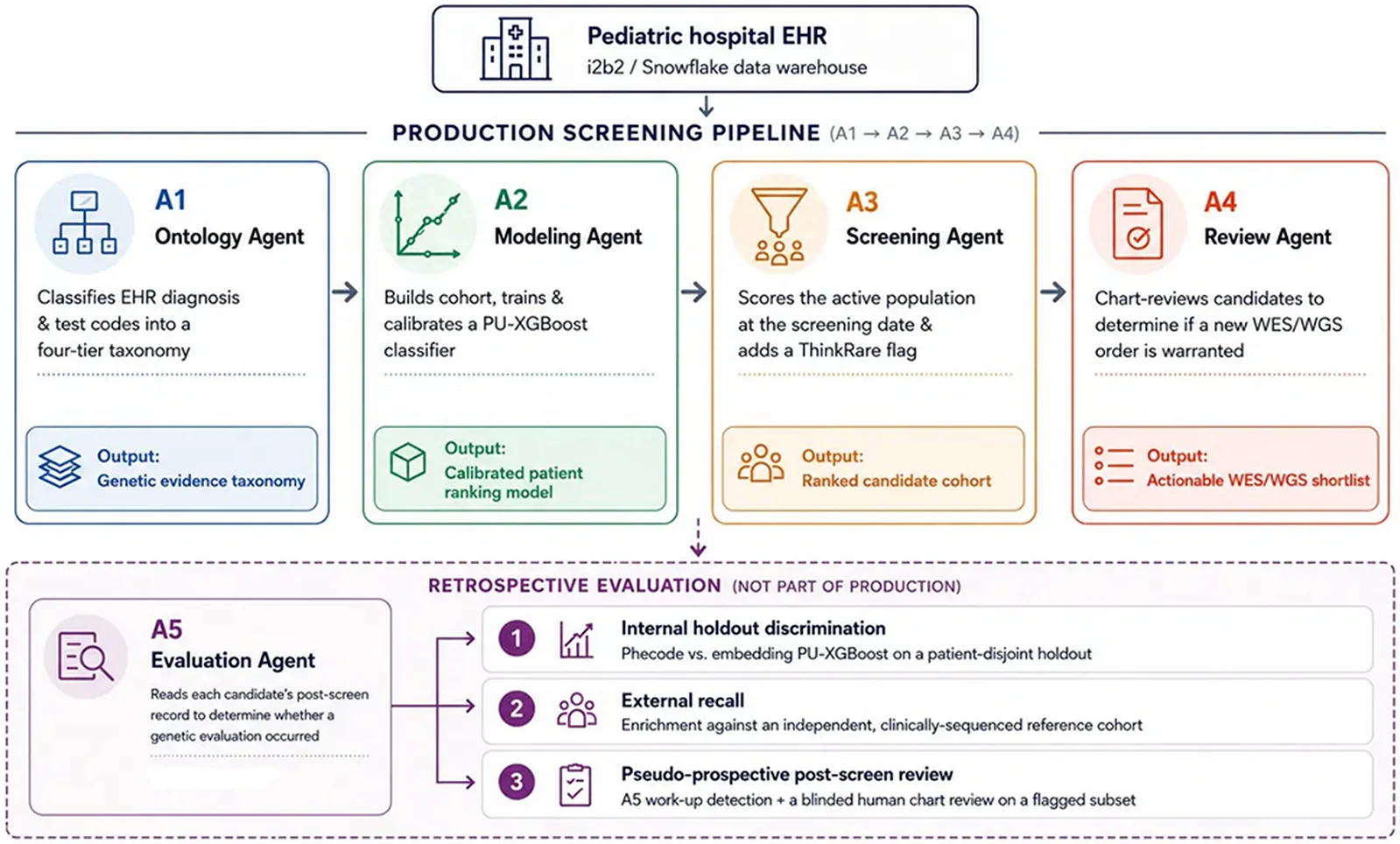
Overview of the multi-agent RESCUE design and evaluation studies The Ontology Agent (A1) classifies EHR diagnosis and test codes into a four-tier genetic-evidence taxonomy; the Modeling Agent (A2) builds a case/control cohort and trains a calibrated PU-XGBoost classifier; the Screening Agent (A3) scores the active patient population at the screening date and adds a rule-based ThinkRare flag; and the Review Agent (A4) chart-reviews candidates to produce the final eligible list for a new ES/GS order. A1–A4 run in production; the Evaluation Agent (A5) is used only retrospectively, to check whether flagged patients later underwent genetic evaluation.

An overview of the multi-agent RESCUE design and evaluation is shown in Figure 1. RESCUE is implemented as four agents (with one additional agent reserved for evaluation in this study; see Evaluation). Each agent has a defined goal, a fixed set of skills, and a single canonical output. Users can control the configuration file (or use another agent called “Vibe Coding” to modify it) to provide details of the parameters, such as screening date and index window. The agents were chained by an orchestra by running in order, with each agent’s output feeding the next. Every long running call is checkpointed row by row with results cached locally after each patient is processed, so an interruption resumes from where it stopped without repeating completed API calls. We provide a brief description of each agent below. The detailed agent settings can be found on https://github.com/stormliucong/RESCUE-n8n.

### Agent A1: Ontology Agent

**Goal:** turn the raw EHR concept dictionary into a four-tier genetic-evidence taxonomy.

**Skills:**

- A Snowflake query to query i2b2 over OBSERVATION_FACT (i.e., the primary table describing all the clinical details of patients) and CONCEPT_DIMENSION (i.e., a dictionary mapping each clinical code (i.e., ICD code, test code) to the specific descriptions) to enumerate every ICD code and every keyword-flagged test concept.
- Instructions and examples to classify each ICD or internal test code into a predefined semantic category based on code description and their metadata.

**Outputs:**

- **Tier 1A** — ICD codes classified as genetic indications (risk-enrichment).
- **Tier 1B** — non-diagnostic / screening / pharmacogenomics-related genetic tests (control-purification).
- **Tier 2A** — test codes classified as diagnostic genetic tests (case-defining).
- **Tier 2B** — ICD codes classified as established genetic diagnoses (case-defining).

### Agent A2: Modeling Agent

**Goal:** assemble a positive-unlabeled cohort with matched controls based on the class labels produced by A1; construct patient feature matrices from pre-index diagnostic history and train a calibrated PU-XGBoost screening model.

**Skills:**

- A Snowflake query to extract 300-character windows around ES/GS keyword mentions in clinical notes and classify whether each mention reflects a rare-disease diagnostic context.
- A Snowflake query to assemble a positive-unlabeled (PU) cohort (cases = Tier 2A/2B or ES/GS-in-context patients; unlabeled pool purified of all Tier 1/2 exposure), with one-to-many matching by age band and per-year visit count.
- Instructions to construct two parallel feature representations from pre-index ICD-10 codes, excluding case-defining Tier 2A/2B codes to prevent leakage: (a) a binary Phecode matrix with visit aggregates and age at index, and (b) dense 3,072-d vectors via text-embedding-3-large applied to a structured serialization of each patient’s diagnostic history.
- A validated Python template to train a PU-XGBoost classifier on both feature sets with a patient-level 80/20 stratified holdout and isotonic calibration.

**Outputs:**

- Cohort for PU learning include a training/validation and holdout sets
- PU trained models and metadata (performance) including a Phecode model and an embedding model.

### Agent A3: Screening Agent

**Goal:** apply the trained model (by default Phecode-based) at a fixed screening date and produce a ranked list of candidate patients, alongside an external rule-based label.

**Skills:**

- A Snowflake query to enumerate the institutional patient base (~5 million) at the screen date and apply the same inclusion filter used during training (no prior Tier 2A (diagnostic genetic test) or Tier 2B (confirmed diagnosis) code; ≥2 visits in each of the 2 pre-screen years).
- A validated Python template to re-extract pre-screen Phecode features under the metadata of trained models (missing columns zero-filled), calls the calibrated PU model, and sorts by calibrated probability.
- Instructions and original paper to implement the modified ThinkRare (v6) rule^18^ as faithfully as possible to its specification.

**Outputs:**

The output is one row per screened patient with raw probability from the model, calibrated probability that estimates the likelihood that the patient whose diagnostic history resembles that of the positive class patients and a Boolean label for thinkrare_positive.

### Agent A4: Review Agent

**Goal:** Determine whether ES/GS would be a necessary clinical action for each candidate from A3’s ranked list. In production, the Review Agent runs only on top-ranked candidates; for the retrospective evaluation, parallel control arms were also processed (see Evaluation).

**Skills:**

- A Snowflake query that returns metadata-only summaries of each candidate’s pre-screen notes.
- A weighted-sampling policy that selects up to 10 notes per candidate, with a fixed high-priority weight (=99) for notes with text that contains any of *genetic, exome, genome, sequencing, chromosome, chromosomal, WES, WGS, microarray*, and an exponential-decay × note-type-boost weight for all other notes.
- A technique to reduce LLM API calls per patient by pulling only the sampled note blobs and concatenating them.
- Instructions to classify note blobs to determine prior genetic testing status, and exclude patients with a prior positive genetic test, prior WES/WGS testing, or multiple unfulfilled genetic test orders (for any reason).
- Instructions to assess medical necessity against ACMG clinical criteria and filter out patients who do not meet them (configurable for evaluation).

**Outputs:**

- A final list of patients for whom WES/WGS was found to be medically necessary, and operationally feasible.

### Agent A5: Evaluation Agent (not for production)

**Goal:** For each candidate produced in A4, read what happened in their chart after the screen date and determine prospectively whether they actually went on to have a genetic evaluation. This is only used to study evaluation purposes.

**Skills:**

- A Snowflake query example to flags candidates with enough follow-up to judge. “Enough” means at least 2 post-screen visits, with the last visit at least 183 days (about 6 months) after the screen date.
- A validated Python template to look for a post-screen genetic workup through two channels: a structured channel (any Tier 2A or 2B observation recorded after the screen date) and an unstructured channel (the LLM reading the patient’s post-screen notes, system prompt P5).
- A validated Python template to sample notes by reusing A4’s weighted sampling but flips the time weight so recent notes are favored (1 - exp(-days/365)), gives genetic-keyword notes a high fixed weight, and caps it at 10 notes per patient.
- Instructions to perform chart review on sampled notes per patient and to answer the question: did the patient get a genetic workup after the screen (P8).

**Outputs:**

- A list of candidates who had a post-screen genetic workup. In the case arm (the patients the screen flagged) these are true positives; in the control arm they are false negatives, since the screen missed them.
- A list of candidates who had enough follow-up but no workup found in either channel. These are sent for manual review in the evaluation study.

### Evaluation

We performed three evaluation experiments to benchmark RESCUE:

1. Internal holdout discrimination. Agent scored the Phecode and embedding PU-XGBoost classifiers on a patient-disjoint 20% holdout. Metrics reported includes AUC (area under the ROC curve), AP (average precision), calibration, and stratified odds ratios (ORs) across cases, risk-enriched patients, and purified controls.
2. To evaluate whether RESCUE recognizes rare-disease patients in the absence of explicit coding, we tested it against the Children’s Rare Disease Cohort^25^ (CRDC), an independently curated registry of patients confirmed to have rare diseases through clinical genomic sequencing. Since the CRDC was outside of the coded EHR data used for RESCUE training, it serves as an external ground truth for recall. We pass this cohort through the same patient-inclusion filter, scored the model on every patient, and measured enrichment of CRDC members across configurable cutoffs.
3. Pseudo-prospective (post 2021-12-31) evaluation, comprising a review agent (A5) that adjudicates whether a candidate actually underwent genetic evaluation after the screen date; and a blinded human chart-review on a subset of cases and controls flagged by A5 as having adequate follow-up but no detected genetic workup after the index date.

## 3. Results

The Ontology Agent classified 14,000 high-prevalence ICD-10 codes and 1,200 keyword-flagged test codes into the four-tier framework. The number of included codes in each tier can be found in Supplementary Appendix Table S1. The training and validation cohort thus came to 6,153 unique cases and 56,799 unique controls using the following inclusion criteria: index date = first qualifying event shifted backward 90 days; index window 2018-01-01 to 2021-12-31; activation requires ≥2 visits in each of 2 lookback and 2 follow-up years. The Modeling Agent trained the Phecode and embedding PU-XGBoost classifiers on the same 50,361 training patients and evaluated them on a 12,591-patient holdout (1,230 cases, 11,361 controls). As shown in Table 1, both models achieved comparable performance (Phecode model: AUC = 0.794, AP = 0.400; Embedding model: AUC = 0.808, AP = 0.417) (Table2; Figure S1). Isotonic calibration left both curves close to the diagonal (Figure S2).

**Figure 2.**
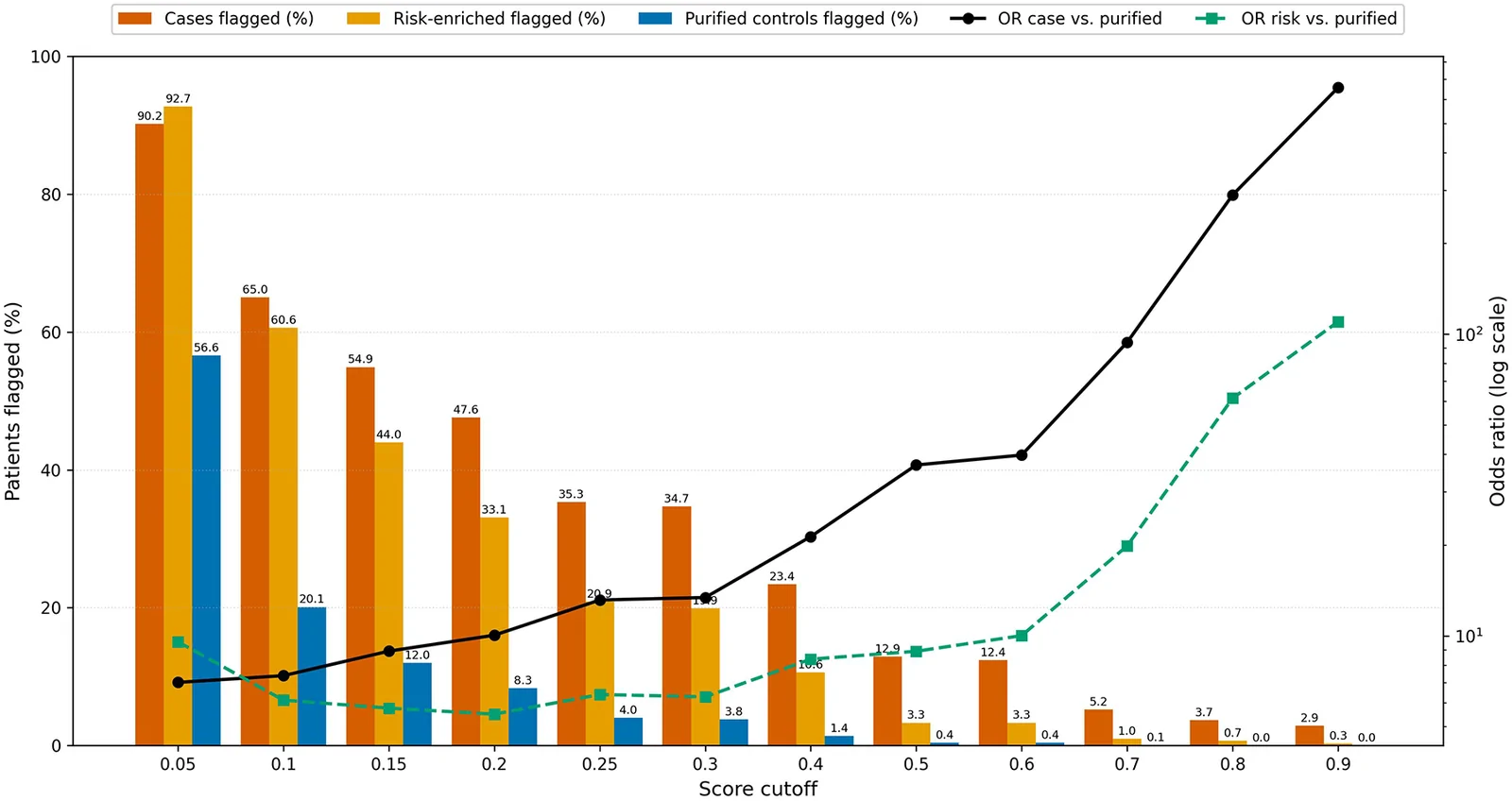
Patients flagged in different groups across model score cutoffs Grouped bars show the percentage of patients flagged (i.e., with a predicted score at or above the cutoff) within each group: cases (vermillion), risk-enriched patients (orange), and purified controls (blue). Data labels above each bar indicate the exact percentage flagged. Overlaid lines show the odds ratio (OR) of being flagged for cases versus purified controls (black, solid) and for risk-enriched patients versus purified controls (green, dashed).

We also checked whether the model picked up patients carrying genetic indication codes (Tier 1A, the risk enrichment tier). At every non-zero cutoff, flagged patients were enriched for these codes (Tables S1 and S2; Figure 2). At a calibrated cutoff of 0.20, for example, the Phecode model flagged 33.1% of patients who had a genetic indication code versus 8.3% of those who did not (OR 10.1).

The Screening Agent then applied the trained models across the full institutional EHR at a screen date of 2021-12-31. Among 494,577 active patients, 97,021 known cases (Tier 2A/B prior to screen; see Table S1) and 268,438 patients with insufficient pre-screen visits were excluded, leaving 175,842 patients meeting the inclusion criteria. The mean calibrated positive prediction probability is 0.077 among those included patients, and the modified ThinkRare (v6) algorithm flagged 2,678 (1.5%) of these patients. Table S3 shows the calibrated-probability cutoff breakdown and the overlap between the PU-trained model and the modified ThinkRare algorithm. Even at probability ≥0.05 only 2.8% of model-positives were ThinkRare-positive, indicating that the two methods carry largely complementary rather than redundant signals.

**Figure 3.**
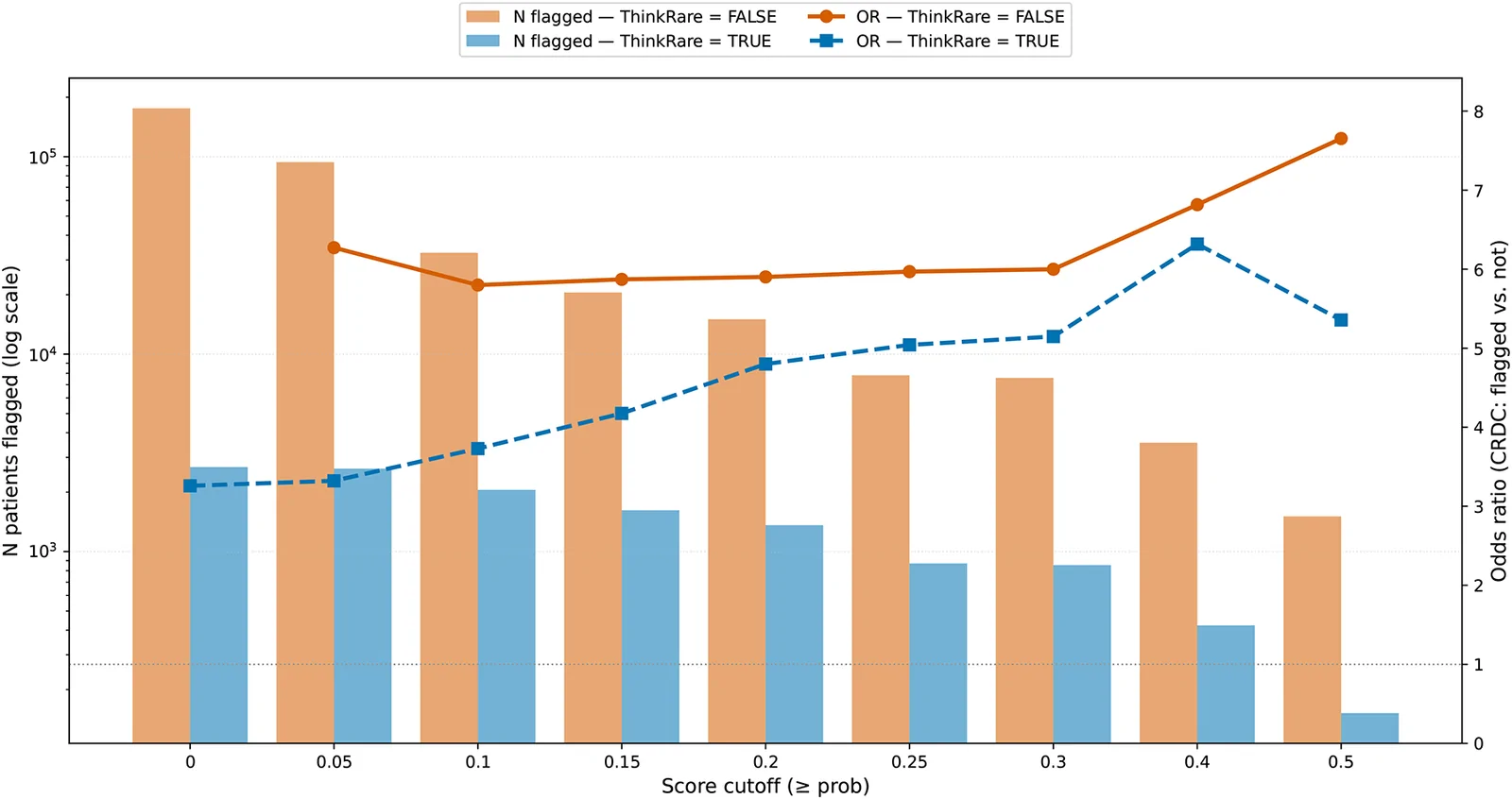
Screening performance based on CRDC cohort Patients were stratified by whether they were independently flagged by ThinkRare (n = 2,678; blue) or not (n = 175,842; vermillion), and the RESCUE model was evaluated separately within each stratum across calibrated prediction probability cutoffs. Bars (left axis, logarithmic scale) show the number of patients flagged at or above each cutoff. Lines (right axis) show the odds ratio of being a confirmed rare disease case (CRDC, patients who have received ES/GS) among flagged versus non-flagged patients at each cutoff; the dotted horizontal line marks an odds ratio of 1 (no enrichment).

We also benchmarked the Screening Agent against the Children’s Rare Disease Cohort (CRDC; n = 8,074 sequenced patients), passing it through the same inclusion filter the agent uses. That left 2,049 CRDC patients eligible at 2021-12-31. At the most enriched setting with probability ≥ 0.20 and ThinkRare-positive, recall was 3.5% and the OR against eligible non-CRDC patients was 4.8. (Table S4; Figure 3).

In production the Review Agent runs only on the top-ranked patients, although this can be customized. For evaluation, we tried four different configurations by pointing it at four comparison arms:

- **M+T+**: calibrated probability >0.5 AND ThinkRare-positive (n = 152).
- **M-T+**: calibrated probability ∈ (0.05, 0.10) AND ThinkRare-positive (n = 577)
- **M+T-**: calibrated probability >0.5 AND ThinkRare-negative,capped at 1,000 random sampled patients.
- **M-T-**: calibrated probability ∈ (0.05, 0.10) AND ThinkRare-negative, capped at 1,000 random sampled patients.

The Review Agent flagged pre-screen prior-testing evidence in 31/152 (20.4%) of M+T+ candidates, 49/577 (8.5%) of M-T+ candidates, 204/1,000 (20.4%) of M+T- candidates, and 32/1,000 (3.2%) of M-T- candidates. After A4 removed candidates with documented prior testing, 121 M+T+, 796 control M+T-, 528 M-T+, and 968 M-T- candidates remained in the operationally actionable lists.

The Evaluation Agent then ran over the follow-up-eligible patients in each arm to see who actually had a post-index genetic workup (Table S5; Figure 6). Patients in the M+T+ arm were 7.4-fold more likely than those in the M-T- arm to have documented genetic-evaluation activity (26.5% vs. 3.6%)

Finally, an independent board-certified genetic counselor did a blinded chart review of a 15-patient subsample for T+M+ and T-M- arm, all flagged by the Evaluation Agent as having no detected post-index workup. In the T-M- arm, the counselor agreed that 14/15 (93.3%) had no genetic relevance or medical necessity to order ES/GS. In the T+M+ arm, 13/15 (86.7%) met medical necessity criteria for testing; one of those had a prior order the Review Agent missed. That left 12/15 (80.0%) T+M+ patients judged both medically necessary and operationally actionable, that is, worth contacting the patient or provider for genomic testing.

## 4. Discussion

Screening electronic health records (EHRs) to identify patients who are appropriate candidates for exome sequencing (ES) or genome sequencing (GS) traditionally involves multiple areas of expertise. This includes EHR coding terminology experts, professionals who can train and validate data-driven models, and genetic specialists who review charts to ensure accuracy and operational feasibility. RESCUE applies this approach by assigning the roles of coding terminology expert, machine learning engineer, and genetic counselor to separate agents, each responsible for one specialized part of the pipeline. By adopting a multi-agent design^26^, each agent is given a narrow and defined scope, making each task easier to specify and the output easier to constrain. The retrospective analysis facilitated by Evaluation Agent (A5) indicates that individuals flagged by RESCUE without prior genetic evaluation had a significantly elevated likelihood of ultimately receiving genetic evaluation and testing. More importantly, a blinded manual review showed that RESCUE successfully identified patients who were previously missed and who would benefit from ES/GS.

Our agentic workflow addresses the limitations of rule-based approaches for identifying rare disease patients, which are difficult to generalize and lack the flexibility to prioritize patients based on each institution’s capacity. We overcome the challenge of the absence of labeled datasets for supervised learning in rare diseases^27^ by employing a positive-unlabeled (PU) learning approach. The Ontology Agent eliminates the need for manual review of institution-specific codes, producing proxy labels for training. Although the actual code may not be easily transferable due to variations in data schemas and internal codes across institutions, the ontology agent and corresponding prompts are expected to be readily adaptable to other clinical settings with minimal changes. Similar to other LLM-powered chart review^28^, The Review Agent adds an operational-feasibility check by sampling the most informative notes to determine if genomic testing has already been ordered and if the patient is operationally reachable. This allows clinically deployed systems to weigh logistical concerns such as ability to contact the patient and family and prior work-up alongside clinical justification. Each clinic can further revise the prompt to encode its own operational rules, enhancing the precision of the flagged list.

Our evaluation demonstrates that the Phecode-based^29^ approach and the embedding-based^30^ approach perform similarly, suggesting that a large transformer-based encoding approach can capture much of the information in human-curated ontology systems. The more operational decision is where to set the screening cutoff, which should be tuned to match each institution’s recontact capacity. A lower threshold maximizes recall at the cost of a larger candidate list, while a higher threshold produces a shorter, more enriched list. In our validation of the classifier’s recall against a rare disease cohort (CRDC) where 2,049 patients are eligible for scoring, the model-only OR among ThinkRare-negative patients remains stable between 5.5 and 6.5 across most cutoffs and rises further at calibrated probability cutoff 0.3. This consistency indicates that the PU-classifier captures a durable rare-disease signal in diagnostic history missed by ThinkRare. Among ThinkRare-positive patients, the OR starts with only 3.3 at cutoff 0, indicating a moderate enrichment by ThinkRare alone, which then climbs steadily to a peak of around 6.5 at cutoff 0.4, suggesting that the addition of a high model probability substantially improves the concentration of true rare-disease patients within the ThinkRare-positive group. These patterns suggest a natural prioritization ladder: when the capacity is limited, institutions may first prioritize the ThinkRare-positive, high-probability intersection for a short, high-confidence list, while those with broader outreach infrastructure can extend to the model-only stratum where both enrichment and candidate volume is higher.

We initially started our design without providing validated Python script or Snowflake query examples and observed that the agent behavior was difficult to control and hard to reproduce^31^. This is likely because human instruction is more ambiguous than actual program codes. To ensure the reproducibility of the agent’s output, we provided agents a set of manually validated query examples as starting scripts. One limitation is that RESCUE has not been tested in across institutions. Cross-site deployment of LLM-based clinical systems faces regulatory barriers, especially different institutional policies on granting HIPAA-compliant LLMs access to patient databases that make multi-site generalization a nontrivial undertaking than a simple replication exercise. Challenges remain in regulating HIPAA-compliant LLMs and governing AI agents that connect to internal clinical data warehouses across different institutions. However, agents should be able to generate institution-specific database queries given institution-specific examples, since the goals are clearly defined in the skills we provided. The framework is also not tied to a single LLM provider and should be adapted to other agentic infrastructure.

## Supplementary Material

Supplement 1

## Figures and Tables

**Figure 4.**
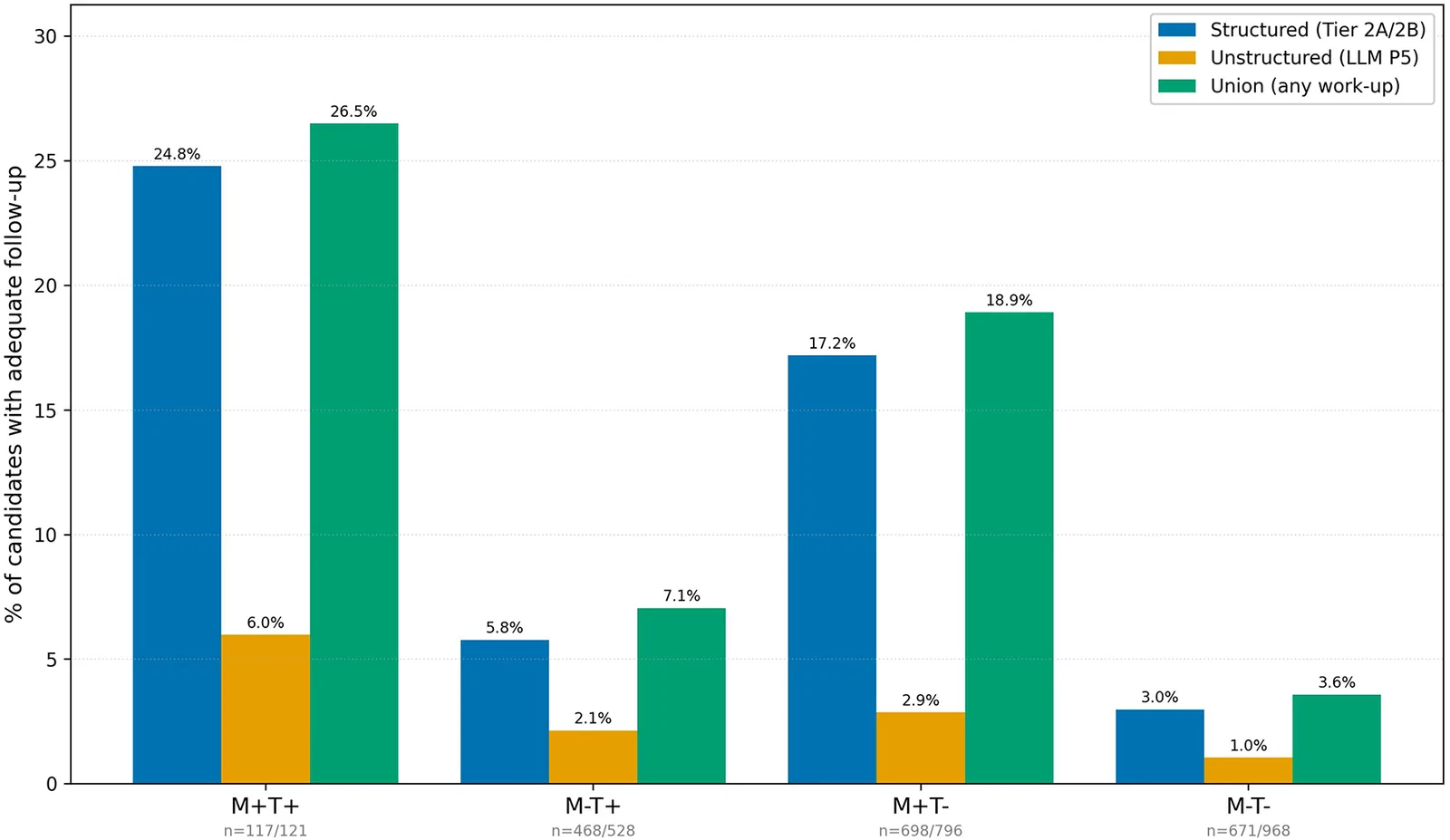
Post-screening evaluation across arms Candidates were partitioned into four mutually exclusive arms by whether they were flagged by the RESCUE model (M+/M−) and whether they were flagged by a rule-based classifier (T+/T−). For each arm, bars show the percentage of candidates with adequate follow-up who had evidence of a diagnostic genetic work-up ordered, by evidence source: structured evidence (Tier 2A/2B diagnostic codes; blue), unstructured evidence (LLM-identified work-up in clinical notes; orange), and the union of any work-up from either source (green). Percentages are expressed relative to the number of candidates with adequate follow-up in each arm.

**Table 1.** Comparison of Phecode and embedding-based PU-classifier on Holdout validation dataset

Model	N features	Per record (Row) AUC	Per record (Row) AP	Patient AUC	Patient AP
Phecode	1,616	0.794	0.400	0.796	0.389
Embedding	3,072	**0.808**	**0.417**	**0.811**	**0.404**

AP: average precision score. AP summarizes a precision-recall curve as the weighted mean of precisions achieved at each threshold, with the increase in recall from the previous threshold used as the weight: AUC: Area Under the Receiver Operating Characteristic Curve (ROC AUC) from prediction scores. Per record: each unique patient in unlabeled group can have more than records (i.e. different index date)

## Data Availability

The data used in this study contain patient-sensitive information and will, therefore, not be published. Requests for access to de-identified data may be directed to the corresponding author and are subject to institutional review and a data use agreement with Boston Children’s Hospital. The source code is available at https://github.com/stormliucong/RESCUE-Agent.
